# Deep Learning for Improving the Effectiveness of Routine Prenatal Screening for Major Congenital Heart Diseases

**DOI:** 10.3390/jcm11216454

**Published:** 2022-10-31

**Authors:** Siti Nurmaini, Radiyati Umi Partan, Nuswil Bernolian, Ade Iriani Sapitri, Bambang Tutuko, Muhammad Naufal Rachmatullah, Annisa Darmawahyuni, Firdaus Firdaus, Johanes C. Mose

**Affiliations:** 1 Intelligent System Research Group, Faculty of Computer Science, Universitas Sriwijaya, Palembang 30139, Indonesia; 2 Internal Medicine, Mohammad Hoesin General Hospital, Palembang 30126, Indonesia; 3 Division of Fetomaternal, Department of Obstetrics and Gynaecology, Mohammad Hoesin General Hospital, Palembang 30126, Indonesia; 4 Department of Obstetrics and Gynaecology, Faculty of Medicine, Padjajaran University, Bandung 45363, Indonesia

**Keywords:** congenital heart disease, classification, deep learning, explainable AI, fetal ultrasound

## Abstract

Early prenatal screening with an ultrasound (US) can significantly lower newborn mortality caused by congenital heart diseases (CHDs). However, the need for expertise in fetal cardiologists and the high volume of screening cases limit the practically achievable detection rates. Hence, automated prenatal screening to support clinicians is desirable. This paper presents and analyses potential deep learning (DL) techniques to diagnose CHDs in fetal USs. Four convolutional neural network architectures were compared to select the best classifier with satisfactory results. Hence, dense convolutional network (DenseNet) 201 architecture was selected for the classification of seven CHDs, such as ventricular septal defect, atrial septal defect, atrioventricular septal defect, Ebstein’s anomaly, tetralogy of Fallot, transposition of great arteries, hypoplastic left heart syndrome, and a normal control. The sensitivity, specificity, and accuracy of the DenseNet201 model were 100%, 100%, and 100%, respectively, for the intra-patient scenario and 99%, 97%, and 98%, respectively, for the inter-patient scenario. We used the intra-patient DL prediction model to validate our proposed model against the prediction results of three expert fetal cardiologists. The proposed model produces a satisfactory result, which means that our model can support expert fetal cardiologists to interpret the decision to improve CHD diagnostics. This work represents a step toward the goal of assisting front-line sonographers with CHD diagnoses at the population level.

## 1. Introduction

Congenital heart disease (CHD) is a structural abnormality of the heart and/or great vessels found prenatally or postnatally [1]. Such conditions cause health problems in millions of babies born every year. It accounts for one-third of all CHDs [1,2]. There are many types of CHD, and they sometimes occur in combination. Some of the more common defects include septal defects, coarctation of the aorta, pulmonary valve stenosis, transposition of the great arteries, and an underdeveloped heart [3]. The top seven CHDs include ventricular septal defect (VSD), atrial septal defect (ASD), atrioventricular septal defect (AVSD), Ebstein’s anomaly (EA), tetralogy of Fallot (TOF), transposition of great arteries (TGA), and hypoplastic left heart syndrome (HLHS) [3,4].

CHD screening can be started at 11 weeks of gestation by using a 4-chamber view (4CV), and sensitivity can increase to 92% at 13 weeks of gestation [5,6]. Through the 4CV, expert fetal cardiologists can ultimately see the cardiac position and its condition in utero [4,6]. If there is an anomaly in the structure and blood vessels of the fetal heart, it can be seen clearly through the 4CV [5]. Many cases of CHD are diagnosed before a baby is born during an ultrasound (US) scan during pregnancy. Advances in fetal echocardiography using high-resolution US and serial imaging have led to an increased number of fetuses diagnosed with CHD. The clinical course in utero and at delivery can now be predicted. Consequently, fetal medicine specialists are asked to consider the fetus as a patient and the transition to postnatal life as a significant part of care [6]. Through US, CHDs can be detected manually. However, it is not always possible to detect CHDs in this way [7,8]. It is a greatly challenging task to analyze such conditions manually due to several key factors, such as numerous speckles in US images, a small fetal heart, unfixed positions, and category indistinction caused by the similarity of fetal heart chambers [9]. Expert fetal cardiologists can detect CHDs with over 98% sensitivity and near 90% specificity [9]. However, a shortage of expert fetal cardiologists means that over 96% of examinations are performed by obstetrics and gynecology generalist [9]. Correspondingly, population-based studies consistently report detection rates of around 40% to 50% [10].

To overcome these challenges, a computer-assisted method helping expert fetal cardiologists to locate and interpret fetal heart anatomy automatically has attracted much attention in recent years [11,12]. Such procedures can help expert fetal cardiologists to support the diagnosis of CHDs with automatic mechanisms. One fetal echocardiography-based computer method is deep learning (DL). Such methods could help close this gap and provide generalist obstetrics and gynecology assistance for difficult diagnoses to increase the prediction rate. The automatic analysis of fetal heart anatomy contributes significantly to the early diagnosis of CHD and the preparation for further therapy. The most successful applications of DL in fetal ultrasounds have been in pre-diagnostic tasks, including standard plane detection [10], the classification and detection of CHDs [13,14,15,16,17,18], and fetal heart developmental assessment [19,20]. We hypothesized that DL could improve the ultrasound analysis of CHDs.

Unfortunately, there have been few studies on diagnostic assistance in fetal heart screening. No research has performed an automatic fetal heart diagnostic process for many CHDs conditions. Most CHD prediction methods use only one or two cases and one control. Furthermore, most previously reported works in the literature had been evaluated based on the intra-patient paradigm rather than the inter-patient scheme, which is a more realistic scenario to prevent training and test the model using samples from the same patients [21]. Therefore, although some of these methods achieved good performance using the intra-patient scheme, their results are unreliable because their evaluation process was biased [17]. To improve the classification approach, this study proposes explainable CNNs to classify, localize, and interpret echocardiograms for distinguishing between normal and CHD conditions. The novelty and contributions of this study are as follows:

- To propose a DL model for classifying eight-class of congenital heart diseases; seven CHDs, such as ASD, VSD, AVSD, EA, TOF, TGA, HLHS, and one control as Normal;
- To extract relevant frames from the normal control (NC) and CHD US video based on an apical four-chamber view (4CV) standard plane;
- To produce the explainable classification result with a combination of gradient activation mapping and guided backpropagation;
- To compare the prediction performance of the proposed model against three expert fetal cardiologist’s interpretations;
- To evaluate the DL model with intra-patient and inter-patient scenarios.

The rest of the paper is structured as follows: Section 2 details our search methodology, and Section 3 presents the results and discussion. Section 4 provides the conclusion.

## 2. Materials and Methods

### 2.1. Data Preparation

The population of this study was 76 pregnant women who presented for routine clinical pregnancy at obstetrics and gynecology, subspecialist fetal cardiology, Mohammad Hoesin General Hospital, Palembang, Indonesia. The subjects in the case group were 31 patients, and there were 45 patients in the normal control group. The fetal heart was examined by taking a four-chamber view (4CV). Table 1 shows that we divided the data distribution into two scenarios based on intra- and inter-patient US to develop a robust model. The whole data comprised about 1129 for training and testing in the intra-patient scenario and about 55 echocardiograms for testing in the inter-patient scenario. The expert fetal cardiologist’s interpretation of the entire US served as our gold standard for the learning process. The dataset represented a real clinical setting well since images were collected prospectively by different operators using GE Voluson E6 machines.

**Figure 1:**
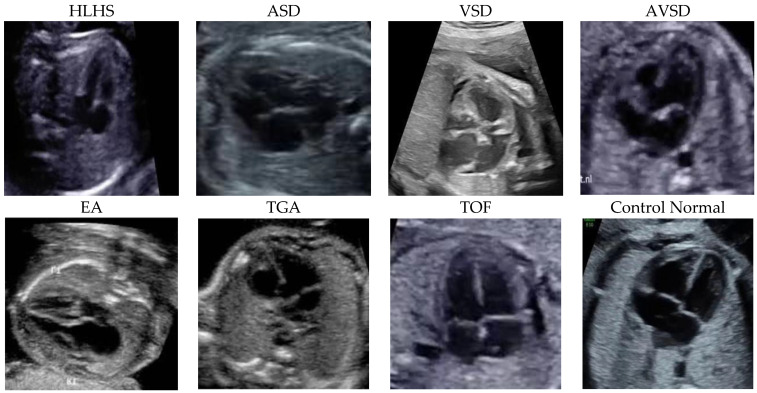
The samples were fetal heart images with seven diseases and a normal control. The entire patient was collected from Mohammad Hoesin General Hospital, Palembang. Indonesia.

Figure 1 depicts the sample of seven diseases, including ASD, VSD, AVSD, EA, TOF, TGA, HLHS, and control echocardiograms from fetal USG. Each patient’s data were collected as raw ultrasound digital imaging and communications in medicine (DICOM) videos. These fetal USG videos are unannotated with precise standard planes, typically used for diagnosis. However, the size of each frame is converted from 640 × 480 pixels to 300 × 300 pixels. Table 1 summarizes the distribution of the frames.

### 2.2. Characteristics of the Research Subject

The general characteristics of the research subjects in each group are shown in Table 2. We used five characteristics—age, body mass index (BMI), trimester, gestation, and parity—to measure the clinical history of each patient before they were predicted based on the DL model. Pregnant women were divided into an age group under or over 35 years, with as many as 31 cases and 45 controls. In the age group above 35 years, there were five cases and 11 controls. In the case group, there were five subjects and 19 subjects who were divided into a BMI of 18.5–22.9 (normal weight) and <18.5 or >22.9 (abnormal weight). In the control group, 12 and 44 subjects were in the normal and abnormal BMI groups, respectively. There were 20 subjects (nine cases and 11 controls) who joined the study in the second trimester (16–26 weeks) and 60 subjects (15 cases and 45 controls) who joined in the third trimester of pregnancy (>26 weeks). Based on gestational history, there were 69 subjects with gravida 1–4 (23 pregnant women in the case group and 46 pregnant women in the control group). One subject in the case group and ten subjects in the control group had a gravida of more than 4. Meanwhile, based on parity, nine nulliparous subjects, and 15 multiparous subjects were in the case group. In the control group, 13 nulliparous subjects, 49 multiparous subjects, and three grandee-multiparous subjects, respectively.

### 2.3. Proposed CHD Classification Architecture

**Figure 2:**
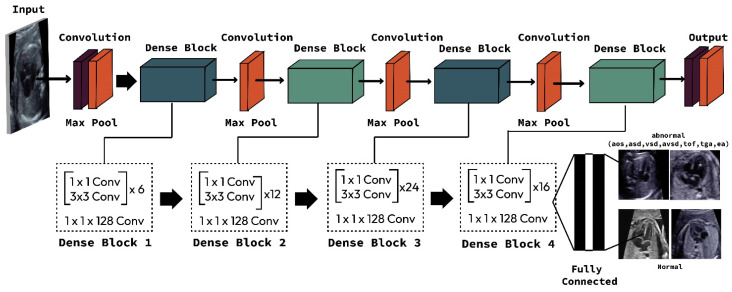
Proposed DenseNet201 architecture to classify seven CHDs and normal control.

**Figure 3:**
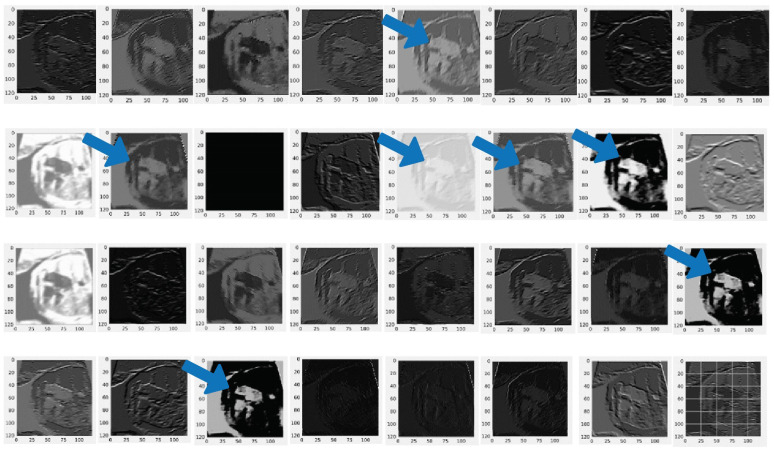
Feature extraction result from convolutional layer with DenseNet201 architecture. Blue arrow indicates the feature generated by from pooling layer in DenseNet 201. The feature maps are the bright areas (indicated by the blue arrow).

This study compared two important CNN architectures, ResNet and DenseNet. One reason that ResNet and DenseNet are exceptionally selected in this study is that the simple design strategy produces good performance [22]. In learning with traditional CNNs, all layers are gradually connected. However, the ResNet architecture proposed employing the shortcut connection by skipping at least two layers. In contrast, the DenseNet architecture offers concatenations of all feature maps from previous layers, which means that all feature maps propagate to later layers and are connected to the newly generated feature maps (Figure 2). CNNs is an end-to-end solution for image classification, it extracts necessary features for efficient image and learns the feature by itself. The feature extraction includes convolution layer piles and sets of pooling layers. As its name implies, the convolution layer transforms the image using the convolution process. It can be described as a series of digital filters. The feature maps are the bright areas (indicated by the blue arrow) are the activated areas, meaning that the filter detects patterns from the given input. This filter captures features of the heart chamber area in the center of the image. The layer of pooling transforms the neighboring pixels into a single pixel and decreases the image dimension. As CNN’s primary concern is the image, the convolution and pooling layers’ procedures are intuitively in a two-dimensional plane. As the sample-generated feature from the DenseNet 201 architecture is presented in Figure 3.

We trained and tested four CNN architectures, including DenseNet121, DenseNet 201, ResNet 50, and ResNet 101. We conducted the learning process without augmentation data to maintain the actual clinical condition. We selected the best model of the CNN architecture from the whole performance. The hyperparameter that controls the learning process is selected with a learning rate of 0.001 based on the SoftMax activation function. To optimize the network, adaptive moment estimation (Adam) is utilized with categorical_crossentropy as the loss function. For reducing overfitting, dropout (0.5) is used as a regularization technique. All the networks were implemented using Python and the PyTorch 1.7.1 library and trained using a computer with specifications as follows: processor with Intel^®^ Core™ i9-9920X CPU @ 3.50GHz, 490,191 MB RAM, and GeForce 2080 RTX Ti, by NVIDIA Corporation GV102 (Rev A1). The operating system was Ubuntu 18.04.5 LTS.

The DenseNet201 model consists of convolutional blocks and two fully connected layers with localization based on gradient-weighted class activation mapping (Grad-CAM) and guided backpropagation (Guided BP) [23]. Such a process explains fine-grained details in the abnormal image. In this study, we combine two techniques to visualize the classification result. The combination produces class discrimination and highlights important fine-grained regions of an echocardiogram for the prediction of CHDs and normal in high resolution for the proposed CNN architecture model. Finally, a combination of Grad-CAM and Guided BP as explainable artificial intelligence (XAI) algorithms are applied to localize and visualize the image features that are most important for the prediction. The internal representations of the specific input for each convolutional layer in the model can be visualized through feature maps. Through the feature map, we can analyze the classification by region.

### 2.4. Model Evaluation

Fetal US based on DL algorithms is evaluated using different metrics according to the addressed task. For classification tasks, performance is assessed using the confusion matrix, with True Positives (TP), True Negatives (TN), False Negatives (FN), and False Positives (FP). The gold standard of classification metrics computed from the confusion matrix is: (1) Accuracy is the number of correct predictions (TP + TN) divided by the total number of predictions (N); (2) Sensitivity is the fraction of actual positives which are correctly identified; (3) Specificity is the fraction of actual negatives which are correctly identified; (4) Negative predictive value in distinguishing normal from CHDs; and (5) Positive predictive value in distinguishing CHDs from normal.

## 3. Results and Discussion

### 3.1. The Classifier Performance

Worldwide, expert fetal cardiologists can detect as few as 30% to 50% of these CHD conditions before birth [24]. However, combining human-performed US and machine analysis allowed researchers to detect 95% CHDs in their test dataset. This work experimented with DL and fetal cardiology experts to observe whether DL could improve fetal CHD detection. We implemented four CNN architectures. However, DenseNet201 results outperformed those of state-of-the-art methods in most of them, and the results provided a classification of CHDs versus normal.

**Figure 4:**
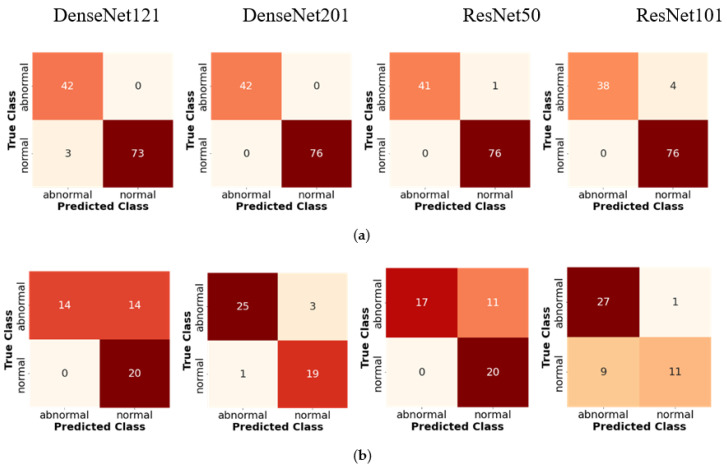
Confusion matrices of two-class classification of four CNN classifier backbones based on intra- and inter-patient scenarios: (**a**) intra-patient validation; (**b**) inter-patient validation.

Table 3 and Table 4 illustrate the classification results for four CNN architectures regarding accuracy, sensitivity, and specificity. These three metrics are the gold standard of the DL model. Satisfactory performance was produced in the intra-patient scenario. However, the DenseNet201 reach had 100% sensitivity (85.75–100%), 100% specificity (93.62–100%), 100% positive predictive value, 100% negative predictive value, and 100% accuracy (95.49–100%; Table 3 and Table 4). The confusion matrix (CM) shows a perfect match between the predictions of the proposed model and the actual image, which means that there was no error prediction in both the cases or controls. When tested with inter-patient data, the performance of the proposed model was maintained, and the sensitivity, specificity, and accuracy values remained above 90%. Comparisons of validation performance and test performance are depicted in the CM (Figure 4). Our DenseNet201 model is significantly better than all four CNN architectures, with the highest performance among all state-of-the-art approaches.

**Figure 5:**
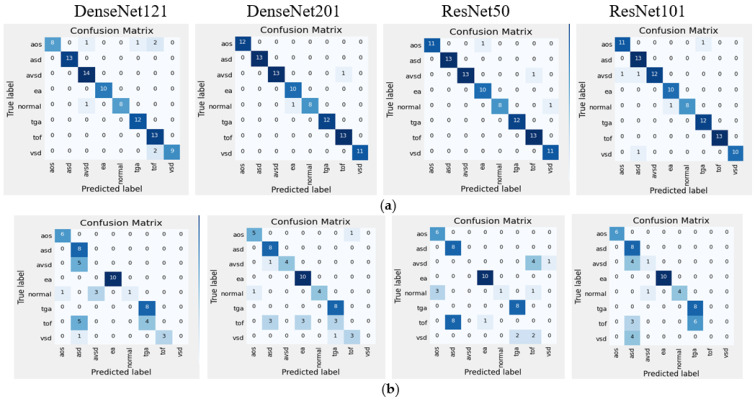
Confusion matrices of eight-class classification of four CNN classifier backbones based on intra-and inter-patient scenarios: (**a**) intra-patient validation; (**b**) inter-patient validation.

In this study, we have also trained, validated and tested the eight-class (seven-class of diseases and normal). The average performance of the classifier with four architectures in intra-patient and inter-patient scenarios is presented in Table 5, and the CM is depicted in Figure 5. It can be seen that DenseNet201’s performance on eight-class classification outperforms other architectures and two-class classification. In intra-patient scenarios, the whole classes can be classified by the DenseNet201 model (Figure 5a). However, in the inter-patient scenario, two classes are unclassified (Figure 5b). The class TOF is misclassified as ASD, EA, and TGA, and the class VSD is misclassified as TOF and TGA. From the validation with inter-patient data using such four architectures, none of them succeeded in recognizing TOF and VSD, due to the two types of diseases affecting each other with similar structural abnormalities. One type of abnormality in the TOF class is the complicated VSD, thus the pixels on the TOF echocardiogram will be identical to the VSD class. This is a big challenge to recognize similar disorders but different diseases. In addition, the limited number of echocardiograms makes the learning process on DenseNet 201 produce an insignificant result.

### 3.2. Improving Classifier Performance by Data Augmentation

**Figure 6:**
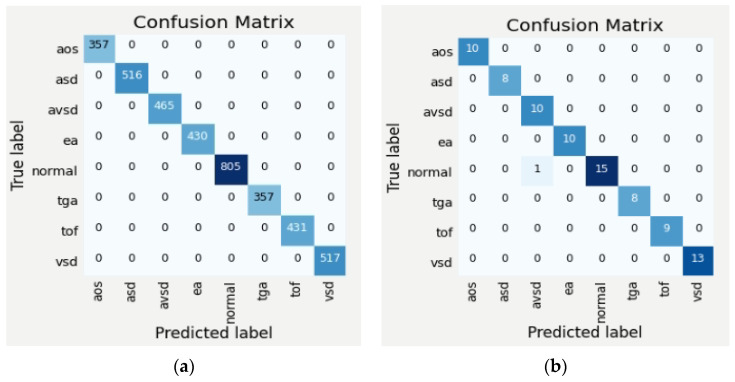
Confusion matrices of eight-class by DenseNet201 model with intra- and inter-patient scenarios after data augmentation (**a**) intra-patient; (**b**) inter-patient.

DenseNet201 performs well from the CM compared to other models (Figure 5). However, such classifier performance in the inter-patient scenario must be improved because there are two classes (TOF and VSD) that fail to be classified due to insufficient data. To overcome such conditions, data augmentation is performed to improve the DenseNet201 model performance. The augmentation process is implemented by using echocardiogram geometric transformation. We serve image rotation (±15 degrees), height and width shift, and vertical and horizontal flipping. We increase the training data from 1129 to 23,504 echocardiograms (19,626 for training and the rest for the validation process). Based on such a process, the classifier performance is increased by 30–35%, especially in the inter-patient scenario (Table 6). In addition, TOF and VSD are classified successfully that previously failed to classify (Figure 5b and Figure 6). For binary and multiclass classification, analyses of CM and incorrectly classified images helped determine that the model error mirrored uncertainties in clinical practice.

### 3.3. Deep Learning against Fetal Expert Cardiologists

To maintain that our model could work robustly in real-world clinical settings, we compared the CHD prediction against three clinicians with expertise in fetal cardiology. One by one, they were shown about 1609 images in the intra-patient set and asked if each image displayed a CHD. The detailed result prediction by expert fetal cardiologists is revealed in Table 7.

**Figure 7:**
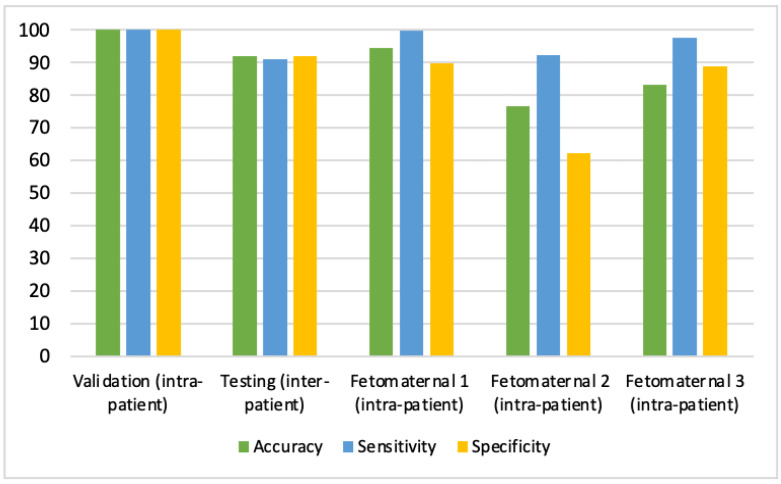
Performance of CHD Prediction Based on Validation (Intra-Patient), Testing (Inter-Patient), and Three Expert Fetal Cardiologists.

Table 7 displays the varying degrees of Kappa values of CHDs interpretations between expert fetal cardiologists and the proposed CNN model. The results of the Kappa test between the first expert fetal cardiologists’ interpretation and the proposed CNNs predictions demonstrated a substantial degree of conformity with the Kappa value of about 0.912, with a 99.73% positive predictive value and a 91.74% negative predictive value. Hence, the first expert fetal cardiologists’ interpretation is almost equal to the proposed CNN prediction. However, the second and third expert fetal cardiologists only reached the Kappa value of about 0.542 (99.73% positive predictive value and 62.23% negative predictive value) and 0.669 (97.52% positive predictive value and 70.31% negative predictive value), respectively. In other words, the expert fetal cardiologist’s interpretation performance undershot CNN’s predictions (Figure 7). The results obtained following previous research using the DL method from fetal US images can help increase the detection of fetal heart abnormalities compared to human experts [10,17,18,24]. Correspondingly, our DL model can support the expert fetal cardiologist’s interpretation of fetal echocardiography US for making diagnoses. In conclusion, our approach to modeling design (intra-patient) and testing (inter-patient) ensured fetal echocardiography US interpretability with satisfactory performance in terms of accuracy, sensitivity, specificity, positive predictive value, and negative predictive value, which can assist clinical adoption.

**Figure 8:**
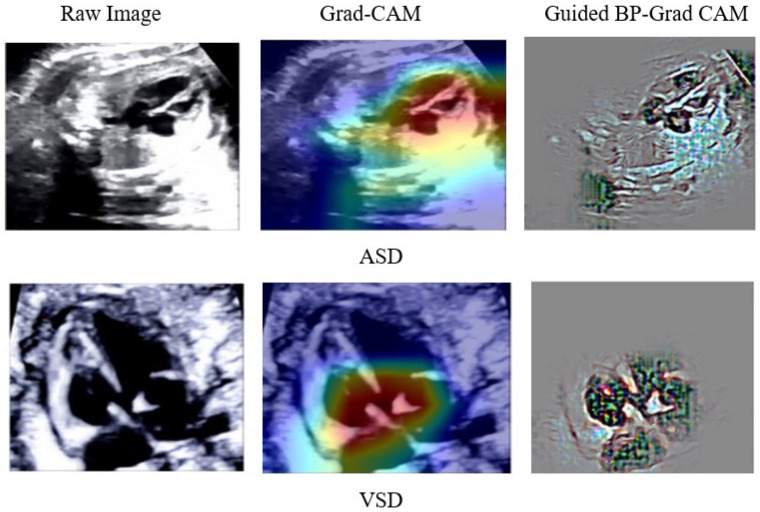

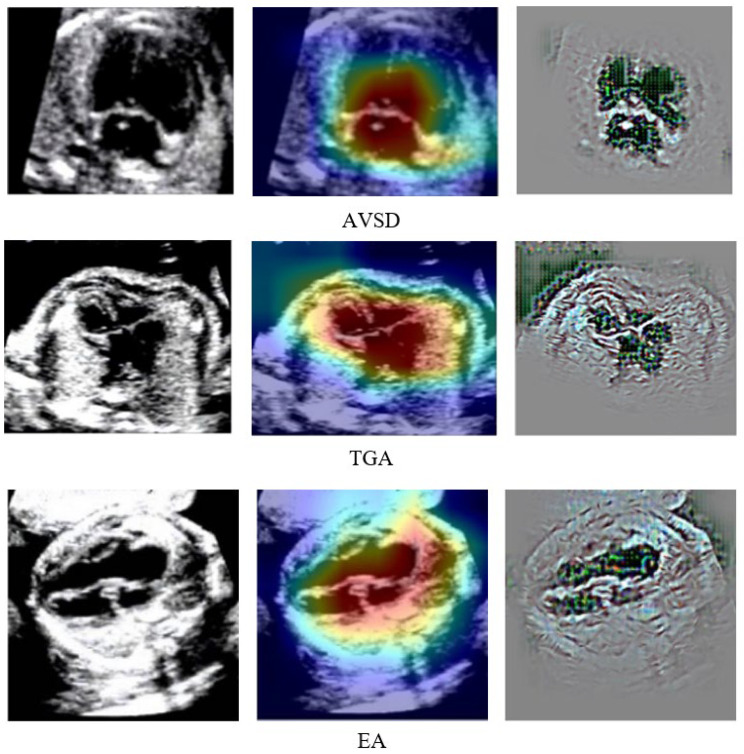

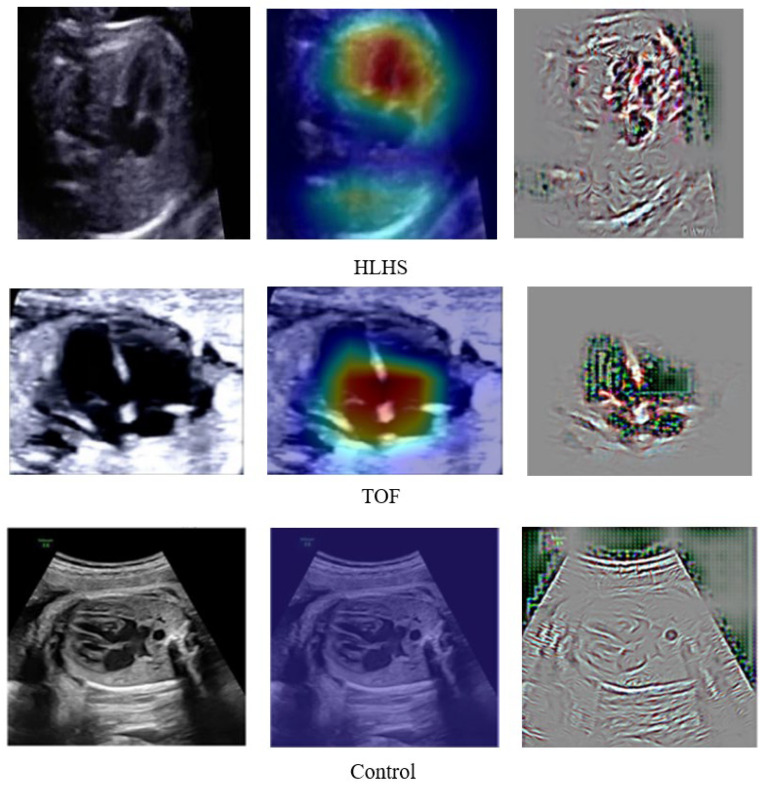
DenseNet201 Classification Result Explained by Guided–BP and Grad–CAM.

To explain the results of DenseNet201 classification so that they can be easily understood medically, we created a visualized image output after the classification process. A combination of guided backpropagation (Guided–BP) and gradient class activation mapping (Grad–CAM) was combined to describe abnormal pixels from US images as CHDs. Combining Guided–BP and Grad–CAM allowed us to generate sharp attributions. Class-discriminative visualization enables expert fetal cardiologists to understand where models are predicted. It can be used for any CNN-based model. A good explainable model should highlight fine-grained details in the image to visually explain why the model predicted a class. Such results can improve expert fetal cardiologists understanding from a medical point of view. Figure 8 depicts the raw image, Grad–CAM, and a combination of Guided–BP and Grad–CAM visualization. There was an excellent localization process for most of the images used in our experiment. Such visualizations can be used to inform downstream model enhancements.

### 3.4. Proposed DenseNet201 Model against State-of-the-Art

To ensure the robustness of the proposed model, we compared the performance of our architecture against several state-of-the-art methods [9,13,14,15]. Table 8 illustrates that our classifier model achieved the best performance in diagnosing fetal CHDs in intra-patient data, with approximately 100% accuracy, sensitivity, and specificity. Hence, our simple yet effective classifier model is more reasonable than other models. To further evaluate the effectiveness of our model, we compared its performance with the model used by [13] on interpatient data. Our proposed DenseNet201 classifier had 92% accuracy, 91% sensitivity, and 92% specificity, whereas the RLDS achieved 91% accuracy and 91% sensitivity. Unfortunately, the RLDS architecture has three classes of CHDs (HRHS, HLHS, and highly RAS) versus the normal control. Correspondingly, our proposed model displays relatively higher accuracy in intra- and inter-patient data and is thus credible enough for the early diagnosis of fetal CHDs.

CHDs are the most common birth defect, and diagnosing them early before birth is very important. They are still rare enough that detecting them is difficult, even for trained clinicians, unless they are highly subspecialized. Such conditions can produce sensitivity, and specificity can be quite low. Early screening with DL may also be helpful for fetal cardiac specialists. Although the performance of fetal echocardiography is excellent in expert hands, there are still potential routes to improve this performance. Due to the small fetal heart size, the dynamics of the fetal position and the fetal heart take up a relatively small proportion of the image, meaning that the DL algorithm must learn to ignore a large proportion of the available data.

## 4. Conclusions

This article proposes a novel, multiple-CHD classification method based on DenseNet201. We have expatiated every step of our method and the following evaluation results. The experiments showed that our method, DenseNet201, is superior to state-of-the-art approaches. The proposed model successfully predicts CHDs with good performance, validated with inter-patient data and three expert fetal cardiologists. While CHD is the most common congenital disability, CHD is still relatively rare.

Moreover, unlike modalities such as photographs, ECGs, or chest X-rays, each ultrasound study contains thousands of image frames. Therefore, designing a DL model to work on many non-independent images from a relatively small subject dataset was an important challenge to overcome. The strengths of our proposed model are the effort to find robust diagnostics for CHDs and the allowed computational efficiency, which is key to translating this work into real-world and resource-poor settings where it is needed. Therefore, prospectively, expanded model testing will be necessary in multiple centers in the future. Several improvements in model algorithms and more training data from more centers may further boost the performance and allow the diagnosis of specific CHD types.

## Figures and Tables

**Table 1:** Distribution of patients and clean cardiac frames.

Number of Data	Seven Diseases	Normal	Total
Unique patient	31	45	76
Frames for training (intra-patient)	812	157	969
Frames for testing (intra-patient)	140	20	160
Frames for testing (inter-patient)	50	5	55

**Table 2:** General characteristics of research subjects.

Normal Control	Cases			
	Frequency (n)		Percentage (%)	
Age				
20–35 year	19	79.17	45	80.36
>35 years	5	20.83	11	19.64
Body Massa index (BMI)				
Normoweight	5	20.83	12	21.43
Abnormal weight	19	79.17	44	78.57
Trimester				
second	13	54.2	22	39.3
third	11	45.8	34	60.7
Gestation				
1–4	23	95.83	46	82.14
>4	1	4.17	10	17.86
Parity				
0	9	37.5	13	23.21
1–4	15	62.5	40	71.43
>4	0	0	3	5.36

**Table 3:** Four Classifier backbones for predicting two-class CHDs.

Metrics	Class	Performance (%)							
		DenseNet121		DenseNet201		ResNet50		ResNet101	
		Intra	Inter	Intra	Inter	Intra	Inter	Intra	Inter
Accuracy	CHDs	97	67	100	93	99	76	95	84
	Normal	98	74	100	90	99	78	97	69
Sensitivity	CHDs	93	100	100	96	98	100	90	96
	Normal	100	59	100	86	100	61	100	55
Specificity	CHDs	100	50	100	89	100	65	100	75
	Normal	96	100	100	95	99	100	95	92

* Intra: intra-patient; Inter: inter-patient.

**Table 4:** Average performance with intra- and inter-patient scenarios.

Metrics	Average Performance (%)							
	DenseNet121		DenseNet201		ResNet50		ResNet101	
	Intra	Inter	Intra	Inter	Intra	Inter	Intra	Inter
Accuracy	97	71	100	92	97	77	97	79
Sensitivity	97	79	100	91	95	80	95	76
Specificity	98	75	100	92	98	82	96	83

* Intra: intra-patient; Inter: inter-patient.

**Table 5:** Eight-class classification average performance with intra and inter-patient scenarios before data augmentation.

Metrics	Average Performance (%)							
	DenseNet121		DenseNet201		ResNet50		ResNet101	
	Intra	Inter	Intra	Inter	Intra	Inter	Intra	Inter
Accuracy	93	60	98	71	97	60	95	67
Sensitivity	87	49	90	62	88	48	89	56
Specificity	90	53	98	68	97	53	95	62

* Intra: intra-patient; Inter: inter-patient.

**Table 6:** Eight-class classification average performance after data augmentation.

Metrics	DenseNet201′s Performance (%)			
	Before Augmentation		After Augmentation	
	Intra-Patient	Inter-Patient	Intra-Patient	Inter-Patient
Accuracy	98	71	100	99
Sensitivity	90	62	100	97
Specificity	98	68	100	98

**Table 7:** Interpretation by expert fetal cardiologists.

Interpretation		Actual Label		Total	Kappa Value
		CHDs	Normal		
Expert 1	CHDs	765 (99.74%)	2 (0.26%)	767	0.912
	Normal	69 (8.27%)	766 (91.73%)	835	
Expert 2	CHDs	709 (92.43%)	58 (7.57%)	767	0.540
	Normal	318 (37.77%)	524 (62.23%)	842	
Expert 3	CHDs	748 (97.52%)	19 (2.48%)	767	0.669
	Normal	250 (29.69%)	592 (70.31%)	842	

**Table 8:** Benchmarking with State-of-the-Art CNNs.

Method	Class	Data Validation	Performance (%)		
			Accuracy	Sensitivity	Specificity
Ensemble Neural Network [9]	2 classes (normal vs. and HLHS)	intra-patient	-	89	92
	2 classes (normal vs. TOF)	intra-patient	-	71	89
Residual learning [13]	2 classes (normal vs. CHDs including HRHS, HLHS, highly RAS)	intra-patient	93	93	-
	2 classes (normal vs. CHDs including HRHS, HLHS, highly RAS)	inter-patient	91	91	-
Deep learning model [14]	2 classes (normal vs. TOF)	intra-patient	-	75	76
	2 classes (normal vs. HLHS)	intra-patient	-	100	90
DGACNN [15]	2 classes (normal vs. CHD)	intra-patient	85	-	-
Proposed	2 classes (normal vs. CHDs including ASD, VSD, AVSD, EA, TOF, TGA, HLHS)	intra-patient	100	100	100
	2 classes (normal vs. CHDs ASD, VSD, AVSD, EA, TOF, TGA, HLHS)	inter-patient	92	91	92
	8 classes (normal, CHDs ASD, VSD, AVSD, EA, TOF, TGA, HLHS)	inter-patient before augmentation	71	62	68
	8 classes (normal, CHDs ASD, VSD, AVSD, EA, TOF, TGA, HLHS)	Inter-patient after augmentation	99	97	98

## Data Availability

The datasets generated and/or analyzed during the current study are available at https://github.com/ISySRGg/LLCNNs (accessed on 1 February 2022). All other data that were generated and analyzed during the current study are included in this published article.
